# Effects of Emotional Intelligence on the Impression of Irony Created by the Mismatch between Verbal and Nonverbal Cues

**DOI:** 10.1371/journal.pone.0163211

**Published:** 2016-10-07

**Authors:** Heike Jacob, Benjamin Kreifelts, Sophia Nizielski, Astrid Schütz, Dirk Wildgruber

**Affiliations:** 1 Department of Psychiatry and Psychotherapy, University of Tübingen, Tübingen, Germany; 2 Department of Psychology, Chemnitz University of Technology, Chemnitz, Germany; 3 Department of Psychology, University of Bamberg, Bamberg, Germany; University of Nottingham, UNITED KINGDOM

## Abstract

Emotional information is conveyed through verbal and nonverbal signals, with nonverbal cues often being considered the decisive factor in the judgment of others’ emotional states. The aim of the present study was to examine how verbal and nonverbal cues are integrated by perceivers. More specifically, we tested whether the mismatch between verbal and nonverbal information was perceived as an expression of irony. Moreover, we investigated the effects of emotional intelligence on the impression of irony. The findings revealed that the mismatch between verbal and nonverbal information created the impression of irony. Furthermore, participants higher in emotional intelligence were faster at rating such stimuli as ironic expressions.

## Introduction

Emotions are involved in daily social interactions, with human beings elaborately applying both verbal (e.g., spoken language) and nonverbal cues (e.g., facial expressions, tone of voice) to express their feelings. These two levels of emotional information can either resonate with each other, leading to congruent signals, or conflict with each other, leading to incongruent signals. When signals are incongruent, nonverbal cues have frequently been reported to have a greater influence on the perception of the counterpart’s emotional state as compared with verbal cues [1–3]. Moreover, a mismatch between the content that is transferred verbally versus nonverbally is one way to generate ironic remarks.

Irony can be defined as a specific “use of words to express something other than and especially the opposite of the literal meaning” [4]. Besides the utilization of contextual information (i.e., standing in the rain while stating “what a beautiful day”), the intended meaning of an ironic message can be encoded as a contrast between linguistic and paralinguistic (nonverbal) signals. In this case, as specified by Anolli and colleagues ([5], p. 155), the decoding of an ironic meaning does not seem to require further contextual knowledge:

In any case, for irony, we have a specific “*contrastive semantic synchrony*”, since the paralinguistic pattern appears to be in contrast to the linguistic input. With the linguistic unit speakers convey a certain meaning obtained by a pure linguistic encoding; with the paralinguistic pattern they convey an alternative (in most cases, opposite) meaning.

A study examining communication among friends revealed that different types of irony were used in 8% of the time the friends spent communicating [6], indicating that irony is not a rare phenomenon but occurs regularly in daily communication. One reason for the regular use of irony could be that in critical situations, irony allows speakers to implicitly address their true feelings, intentions, and attitudes, but at the same time, to comply with social norms [7] and, for example, avoid harsh criticism. Observing proprieties at the verbal level gives speakers the opportunity to subsequently withdraw their “nonverbally honest” information by repeating the same verbal message with a different, non-conflicting tone of voice and facial expression [7]. Irony can thus be used to weaken criticism and protect the relationship [8–9].

The complexity of irony requires the communicative competencies of not only the speaker but also the recipient [5]. To decode irony, the perceiver must be able to discriminate between the literal and the intended meaning of a message. This raises the question of the possible components of communicative competencies that promote the deciphering of ironic messages. Recent studies have revealed positive effects of empathy on the comprehension of irony in children [10] and adults [11]. Since empathy can be considered a specific aspect of emotional intelligence (EI), it can be assumed that EI may help in the detection of irony. According to Mayer, Salovey, and Caruso ([12], p. 210), a “high EI individual, most centrally, can better perceive emotions, use them in thought, understand their meanings, and manage emotions better than others. Solving emotional problems likely requires less cognitive effort for this individual.” In addition, our own results revealed that individuals with high EI tended to rely more on nonverbal signals, and moreover, higher EI was related to smaller reaction time differences between emotionally incongruent and congruent stimuli [3]. Thus, individuals with high EI should also have a higher awareness of the important function of nonverbal cues in emotional communication as conveyers of “true” feelings, intentions, and attitudes. Considering that nonverbal cues are used as visual or vocal markers of irony [13–15], persons who are better at perceiving such cues may more easily detect irony and understand the communicative intentions of the speaker more accurately and more quickly.

The main aim of the study was to evaluate whether (and to what extent) the mismatch between verbal and nonverbal cues would create the impression of irony in the perceiver even in the absence of any further contextual information and to determine how this would be related to perceivers’ EI. To this end, we used audiovisual stimulus material comprised of videos with actors conveying positive, neutral, or negative verbal and nonverbal information about their current emotional state [3]. We chose a positive emotion, namely happiness, and a negative emotion, namely anger, because these are two basic emotions that have high relevance in daily communications [16–17]. Moreover, happiness and anger are comparable in terms of being high arousal emotions [18]. Besides, at least two different emotional categories are required to create a strong mismatch between verbal and nonverbal cues (strongly incongruent stimuli: happy_verbal_ + angry_nonverbal_ and angry_verbal_ + happy_nonverbal_; Table 1). In addition, we chose neutral stimuli to create a moderate mismatch between verbal and nonverbal cues (slightly incongruent stimuli: happy_verbal_ + neutral_nonverbal_, angry_verbal_ + neutral_nonverbal_, neutral_verbal_ + happy_nonverbal_, neutral_verbal_ + angry_nonverbal_; Table 1). After presentation of each stimulus, participants were asked to categorize their impression of the speaker’s expression by applying a forced-choice scale that offered the labels “ironic”, “ambivalent”, “angry”, and “happy”. The category “ironic” was chosen to assess the impression of irony. The category “ambivalent” was selected to assure that incongruent stimuli were not simply rated as “ironic” due to a lack of alternatives. The term “ambivalent” was understood as the authentic expression of mixed feelings (e.g., being happy and angry at the same time). We argue that irony and ambivalence are both characterized by an inherent incongruence, with only irony additionally offering a specific rhetorical function beyond the expression of the current emotional state. The categories “angry” and “happy” were chosen to enable participants to select one of the basic emotions conveyed by the verbal or nonverbal cues. Subsequent to the experiment, EI was assessed with the performance-based Mayer-Salovey-Caruso Emotional Intelligence Test (MSCEIT; [19], German version: [20]). To sum up, we tested three hypotheses in this study.

**Table 1:** Overview of the different stimulus combinations.

Verbal valence	Nonverbal valence		
	Positive	Neutral	Negative
Positive	congruent (16)	slightly incongruent (12)	strongly incongruent (12)
Neutral	slightly incongruent (12)	congruent (16)	slightly incongruent (12)
Negative	strongly incongruent (12)	slightly incongruent (12)	congruent (16)

*Note*: Numbers in parentheses are the respective number of stimuli.

1. The mismatch between verbal and nonverbal emotional cues about the current emotional state of the speaker can create the impression of irony in the absence of any further contextual information. We thus hypothesized that incongruent stimuli would be perceived as “ironic” more often than congruent stimuli.
2. With regard to emotional competencies influencing the impression of irony, we hypothesized that EI would be positively associated with the tendency to classify incongruent stimuli as “ironic” and negatively associated with the reaction time needed to make this decision.
3. We hypothesized that when stimuli were incongruent, participants would base their judgment predominantly on the nonverbal part of the message if they chose one of the basic emotions (“angry”, “happy”) and that EI would be positively associated with this nonverbal dominance.

## Materials and Methods

### Participants

Twenty healthy volunteers (10 female; mean age = 24.65 years, *SD* = 3.18 years) participated in the study. All individuals were native German speakers; 18 of them were right-handed, and two were ambidextrous as assessed with the Edinburgh Handedness Inventory [21]. All of them reported normal hearing and normal or corrected-to-normal vision. None of the participants reported a history of neurological or psychiatric illnesses or a history of substance abuse, and they claimed to be without medication.

### Ethics statement

The study was performed in accordance with the ethical principles expressed in the Code of Ethics of the World Medical Association (Declaration of Helsinki), and the paradigms and protocol employed in this study were reviewed and approved by the Ethics Committee at the Faculty of Medicine of the Eberhard Karls University and the University Hospital Tübingen. All participants gave their written informed consent prior to their inclusion in the study and received a small financial compensation for their participation.

### Stimulus material, task, and procedure

To prepare the appropriate stimulus material, four pre-studies were conducted [22]. First, 94 written sentences expressing the speaker’s current emotional state were evaluated with respect to their frequency of use in daily life. Second, the perceived valence of the expressed state was assessed. Based on these ratings, six sentences were chosen, two expressing a neutral (“Ich bin ruhig”/“I am calm”, “Ich bin etwas aufgeregt”/“I am a bit excited”), two a positive (“Ich fühle mich gut”/“I feel good”, “Ich fühle mich großartig”/“I feel great”), and two a negative (“Ich fühle mich unwohl”/“I feel uncomfortable”, “Ich fühle mich erbärmlich”/“I feel awful”) emotional state in the speaker. Next, audiovisual recordings were made of ten professional actors speaking the six sentences with either a neutral, positive (happy), or negative (angry) facial expression and tone of voice. After the stimulus materials were edited, the valences of the nonverbal signals were rated in a third pre-study. Moreover, in a fourth pre-study, the authenticity of the actors’ portrayals was tested. The data assessed in the pre-studies were used to select stimuli with comparable verbal and nonverbal valences and with high authenticity ratings (larger than 4 on a 9-point scale), leading to a final set of 120 videos. For further details on the recordings, editing, pre-testing, selection, and compilation of the stimulus material, please refer to Jacob and colleagues [22].

The final stimulus material consisted of 120 short videos (mean duration = 1,458.67 ms, *SD* = 316.62 ms) depicting the faces of ten professional actors (5 female) while speaking short sentences in German. Both the verbal and nonverbal cues conveyed either positive, neutral, or negative affective information about the speaker’s emotional state. The different combinations led to congruent (i.e., the verbal and nonverbal information had the same valence; 48 of the 120 stimuli; Table 1), slightly incongruent (i.e., the verbal information had a neutral valence, the nonverbal information had an emotional valence, and vice versa; 48 of the 120 stimuli; Table 1), and strongly incongruent stimuli (i.e., the verbal and nonverbal information had contrasting emotional valences; 24 of the 120 stimuli; Table 1). The decision to use unequal numbers of stimuli for congruent, slightly incongruent, and strongly incongruent stimuli was based on the results reported in a study by Trimboli and Walker [23]. The authors varied the camouflaging of the stimuli from 0% (i.e., low camouflage: all messages comprised conflicting verbal and nonverbal information), 50% (half of the messages were congruent), 83% (the majority of the messages comprised consistent verbal and nonverbal information), to 94%. The results revealed that when the camouflaging was low, nonverbal dominance occurred, whereas when the camouflaging was high, nonverbal dominance disappeared. Thus, using too many inconsistent messages may bias the results in such a way that participants easily and quickly figure out the intention of the experimenter and—as a consequence—adjust their responses to satisfy the experimenter’s expectations. Because our interest was in nonverbal dominance, we considered this important finding by Trimboli and Walker [23] and made sure that only a small number of the stimuli were strongly incongruent (24 of 120 stimuli = 20%) and that the vast majority were congruent (48 of 120 stimuli = 40%) and slightly incongruent (48 of 120 stimuli = 40%).

The experiment was run on a computer using the software ‘Presentation’ (Neurobehavioral Systems Inc., Albany, CA, USA). The sound was played through Sennheiser HD 515 headphones (Sennheiser electronic GmbH & Co. KG, Wedemark-Wennebostel, Germany) at a comfortable listening level. The 120 videos were split equally into two blocks balanced across actors, sentences, and emotions. The block order was balanced across individuals, and the stimulus order within each block was randomized. After presentation of each stimulus, participants were asked to categorize their impression of the speaker’s expression. Answers were given on a rating scale that offered the following four response categories: “ärgerlich” (“angry”), “freudig” (“happy”), “ironisch” (“ironic”), and “zwiespältig” (“ambivalent”). To ensure that participants each had the same definitions of the four categories in mind, a brief definition was given for each category and discussed with the participants before the main experiment (“ärgerlich”/“angry”: “Der/die Sprecher/in drückt einen negativen Gefühlszustand aus. Er/sie ist schlecht gelaunt, ärgerlich.”/“The speaker is expressing a negative emotional state. He/she is bad-tempered, angry.”; “freudig”/“happy”: “Der/die Sprecher/in drückt einen positiven Gefühlszustand aus. Er/sie ist gut gelaunt, fröhlich.”/“The speaker is expressing a positive emotional state. He/she is good-tempered, happy.”; ironisch/ironic: “Der/die Sprecher/in verstellt sich, aber er/sie erwartet, dass die wahre Bedeutung seiner/ihrer Äußerung verstanden wird. Die Verstellung wird dabei eingesetzt, um eine besondere Wirkung zu erreichen.”/“The speaker is dissembling his/her feelings, but he/she expects that the true meaning of his/her statement will be understood. The dissemblance is used to produce a special effect.”; “zwiespältig”/“ambivalent”: “Der/die Sprecher/in drückt einen gemischten Gefühlszustand aus. Er/sie erlebt gleichzeitig widersprüchliche Gefühle, die beide vermittelt werden.”/“The speaker is expressing a mixed emotional state. He/she is experiencing concurrent contradictory feelings that are both conveyed.”). In order to avoid effects that could be attributed to the arrangement of the response categories, 20 individual scales were used, each having a different arrangement of the four response categories. Participants were asked to indicate their subjective judgment as quickly as possible via one of four buttons on a Cedrus RB-730 Response Pad (Cedrus Corporation, San Pedro, CA, USA). Answers were expected within a time frame of five seconds, beginning with the stimulus onset. Subsequent to the end of each stimulus, the scale and the answer given by the participants were shown. To acquaint the participants with the use of the response pad and to adjust the volume to a comfortable listening level, a short test run was conducted before the main experiment. The ten practice trials did not include any of the stimuli used in the main experiment.

### Measures of emotional intelligence

The assessment of EI took place with the help of the German version of the MSCEIT [20], a performance-based measure that draws on the definition of EI proposed by Mayer and Salovey [24]. The MSCEIT contains 141 items that represent eight tasks, two for each of the four branches of EI:

1. Perceiving emotions: Participants were asked to identify emotions expressed in pictures of (A) faces and (B) landscapes as well as abstract designs. Participants had to indicate the extent to which the different emotions were expressed in the actual picture. Answers were given on a 5-point scale ranging from 1 (*No/Not at all*) to 5 (*Extreme/Very strong*).
2. Using emotions: Participants were asked (A) to state how helpful different emotions are for solving a given problem and (B) to imagine certain emotional sensations and to match them to non-emotional vocabulary. Responses were made on a 5-point scale ranging from 1 (*Not useful/Not alike*) to 5 (*Useful/Very much alike*).
3. Understanding emotions: Participants were asked to indicate (A) how emotions change over time and (B) how blends of emotions result in complex feelings. Responses were made by choosing one of five possible answers.
4. Managing emotions: Participants were asked to rate the effectiveness of different strategies for managing (A) their own emotions and (B) the emotions of others. Responses were made on a 5-point scale ranging from 1 (*Very ineffective*) to 5 (*Very effective*).

A total EI score was calculated by applying the consensus scoring method. Accordingly, MSCEIT scores reflect the extent to which the participants’ responses match those of a German normative sample (*N* = 3,653, mean age = 29.04 years, *SD* = 10.89 years; [20]). The split-half reliability coefficient for the MSCEIT was .84 for the total EI. In an exploratory approach, the scores on the four branches of the MSCEIT were calculated individually by applying the consensus scoring method.

### Data analysis

The data were analyzed with the software package IBM SPSS Statistics Version 22 (IBM Corporation, Armonk, NY, USA). Categorical ratings as well as reaction times assessed in the computer experiment and the EI scores assessed with the help of the MSCEIT [20] were used as outcome variables. The categorical ratings were transformed into choice frequencies. The Wilcoxon signed-rank test was used to compare differences in non-normally distributed data, and the effect size was calculated with the following formula: *r* = *Z* / SQRT(*N*). When the data were normally distributed, Pearson’s correlation coefficient was used to describe linear relations with EI. When the data were non-normally distributed, Spearman’s rho was used to describe linear relations with EI. When we tested one of the three hypotheses, one-tailed tests were applied, whereas two-tailed tests were used for all exploratory investigations.

As a first step in the data analysis, the choice frequencies and reaction times for the categories “angry”, “happy”, “ironic”, and “ambivalent” were calculated separately for the congruent, slightly incongruent, and strongly incongruent stimuli across subjects. Testing the assumption of normality revealed that the mean choice frequencies for the categories “angry” (*D*(20) = .23, *p* = .01) and “happy” (*D*(20) = .27, *p* < .001) were not normally distributed for the strongly incongruent stimuli, whereas the mean reaction times for all four categories were normally distributed for all three congruence conditions (all *p*s > .05). Thus, additional data analyses involving the choice frequencies were based on nonparametric statistics, whereas additional data analyses involving the reaction times were based on parametric statistics.

Next, to test the first hypothesis, the choice frequencies for the “ironic” category for all three congruence conditions were tested for differences, and the corresponding effect sizes were calculated. Then, to test the second hypothesis, the mean choice frequencies and reaction times for the “ironic” category for all three congruence conditions were correlated with total EI. The mean choice frequencies and reaction times for the “ironic” category for all three congruence conditions were also evaluated for correlations with each of the four branches of the MSCEIT in an exploratory approach. Moreover, the mean choice frequencies and reaction times for the categories “angry”, “happy”, and “ambivalent” for all three congruence conditions were correlated with total EI as well as with the four branches of the MSCEIT in an exploratory approach.

To test the third hypothesis, it was necessary to transform the data. Two new categories were created, one labeled “pro verbal” and the other labeled “pro nonverbal”. The “pro verbal” category comprised all responses in which the verbal information was positive and the chosen category was “happy” or in which the verbal information was negative and the chosen category was “angry”. The “pro nonverbal” category comprised all responses in which the nonverbal information was positive and the chosen category was “happy” or in which the nonverbal information was negative and the chosen category was “angry”. Then, the mean frequencies for the “pro verbal” and “pro nonverbal” judgments were calculated separately for the slightly and strongly incongruent stimuli. Next, nonverbal dominance was calculated separately for the slightly and strongly incongruent stimuli by applying the following equation:

Nonverbal Dominance [%] = Pro Nonverbal / (Pro Nonverbal + Pro Verbal)×100

Two participants never chose the categories “angry” or “happy” when evaluating the strongly incongruent stimuli (but instead used only the labels “ironic” or “ambivalent” in these instances). Thus, it was not possible to calculate the categories “pro verbal” and “pro nonverbal” or nonverbal dominance for these participants. Therefore, the data of these two participants were excluded from the calculation of nonverbal dominance. For the other participants, nonverbal dominance could range from 0%, indicating that the participants’ choice of emotional categories was based solely on verbal information when they evaluated the incongruent stimuli, to 100%, indicating that it was based purely on nonverbal information in these instances. A test of the assumption of normality revealed that the nonverbal dominance calculated for strongly incongruent stimuli was not normally distributed (*D*(18) = .29, *p* < .001). Thus, additional data analyses involving nonverbal dominance were based on nonparametric statistics. Then, to test the third hypothesis, nonverbal dominance calculated for slightly and strongly incongruent stimuli were first tested for differences, and the corresponding effect sizes were calculated. Second, they were correlated with total EI. In an exploratory approach, nonverbal dominance calculated for slightly and strongly incongruent stimuli was also correlated with the four branches of the MSCEIT.

## Results

### Choice frequencies and reaction times

The choice frequencies and reactions times for the categories “angry”, “happy”, “ironic”, and “ambivalent” for the three congruence conditions are summarized in Table 2.

**Table 2:** Choice frequencies and reaction times for the four categories across the three congruence conditions.

	Choice frequencies [%]			Reaction times [ms]		
	*M*	*SD*	*Mdn*	*M*	*SD*	*Mdn*
*Congruent*						
Angry	37.32	5.40	35.49	2573.29	418.22	2694.64
Happy	29.00	5.30	29.03	2153.16	365.31	2141.81
Ironic	9.73	7.41	8.61	3236.40	635.06	3097.86
Ambivalent	23.94	5.87	23.92	3219.03	496.03	3253.64
*Slightly incongruent*						
Angry	34.19	9.64	33.33	2721.46	411.92	2762.87
Happy	16.92	5.49	17.89	2593.27	616.02	2516.03
Ironic	18.68	9.62	17.71	3131.14	529.98	3031.32
Ambivalent	30.21	9.34	29.17	3196.31	434.62	3215.81
*Strongly incongruent*						
Angry	21.42	10.20	21.29	2764.96	549.76	2945.02
Happy	10.24	13.82	4.17	2854.14	916.64	2770.93
Ironic	44.41	20.89	50.00	2979.99	545.19	2913.78
Ambivalent	23.92	14.61	21.29	3060.24	474.23	3017.03

### Emotional intelligence

The participants’ total EI scores ranged from 77.00 to 133.00 (*M* = 109.55, *SD* = 16.25, *Mdn* = 110.00). Their scores on the four branches of the MSCEIT are summarized in Table 3.

**Table 3:** Emotional intelligence assessed with help of the MSCEIT.

MSCEIT	Min	Max	*M*	*SD*	*Mdn*
Total EI	77.00	133.00	109.55	16.25	110.00
Perceiving emotions (Branch 1)	84.00	123.00	107.40	12.19	111.00
Using emotions (Branch 2)	55.00	125.00	101.80	20.20	105.00
Understanding emotions (Branch 3)	74.00	127.00	109.90	12.50	111.00
Managing emotions (Branch 4)	89.00	123.00	106.60	9.94	109.50

MSCEIT = Mayer-Salovey-Caruso Emotional Intelligence Test [20].

### Choice frequencies for the “ironic” category and emotional intelligence

**Fig 1:**
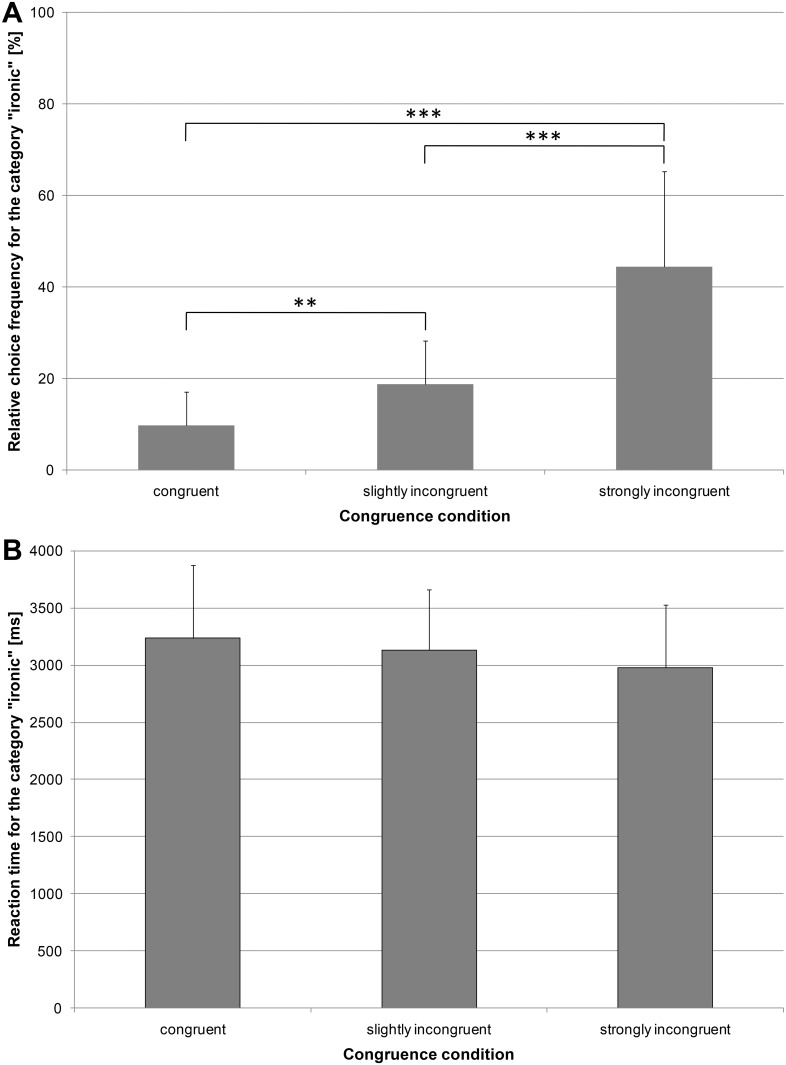
Classification as “ironic” expressions. Bars represent (A) the mean choice frequencies and (B) mean reaction times for the three congruence conditions. Error bars represent the standard errors of the means (*N* = 20). Significant differences are marked with asterisks (*** *p* < .001, ** *p* < .01).

For the choice frequencies for the “ironic” category, congruent stimuli were least often rated as “ironic” (*M* = 9.73%, *SD* = 7.41%, *Mdn* = 8.61%), whereas 18.68% (*SD* = 9.62%, *Mdn* = 17.71%) of the slightly incongruent stimuli and 44.41% (*SD* = 20.89%, *Mdn* = 50.00%) of the strongly incongruent stimuli were rated as “ironic” (Fig 1A). A Wilcoxon signed-rank test showed that the choice frequencies for the category “ironic” for congruent stimuli differed significantly from those for the slightly (*Z* = -3.18, *p* = .001, *r* = -.71, two-tailed; Fig 1A) and strongly incongruent stimuli (*Z* = -3.66, *p* < .001, *r* = -.82, two-tailed; Fig 1A), and the two incongruent conditions also differed significantly from each other (*Z* = -3.32, *p* < .001, *r* = -.74, two-tailed; Fig 1A).

No significant correlations were found between total EI and the mean choice frequencies for the “ironic” category calculated for the congruent (*r*_s_ = .09, *p* = .36, one-tailed), slightly incongruent (*r*_s_ = .07, *p* = .38, one-tailed), and strongly incongruent stimuli (*r*_s_ = -.13, *p* = .29, one-tailed). The correlations between the four branches of the MSCEIT and the mean choice frequencies for the “ironic” category for all three congruence conditions are summarized in Table 4. Moreover, the correlations between total EI as well as the four branches of the MSCEIT and the mean choice frequencies for the categories “angry”, “happy”, and “ambivalent” for all three congruence conditions are summarized in Table 4.

**Table 4:** Spearman’s rho values between emotional intelligence and the mean choice frequencies for the four categories across the three congruence conditions.

	MSCEIT				
	Total EI	Perceiving emotions (Branch 1)	Using emotions (Branch 2)	Understanding emotions (Branch 3)	Managing emotions (Branch 4)
*Congruent*					
Angry^2^	-.07	-.02	.00	-.15	-.18
Happy^2^	.04	.14	.05	.18	-.11
Ironic^1^	.09	.05	-.03	.10	.23
Ambivalent^2^	-.06	-.15	-.07	-.04	-.05
*Slightly incongruent*					
Angry^2^	-.11	.04	-.23	.03	-.15
Happy^2^	-.20	-.04	-.26	.17	-.36
Ironic^1^	.07	-.04	.19	-.12	.19
Ambivalent^2^	.23	.07	.31	-.13	.28
*Strongly incongruent*					
Angry^2^	.19	.24	-.13	.20	.21
Happy^2^	-.12	-.00	-.30	.01	-.13
Ironic^1^	-.13	-.30	.14	-.29	-.13
Ambivalent^2^	-.11	-.07	-.06	.23	-.07

MSCEIT = Mayer-Salovey-Caruso Emotional Intelligence Test [20].

^1^ one-tailed.

^2^ two-tailed.

### Reaction times for the “ironic” category and emotional intelligence

For the reaction times for the “ironic” category, the strongly incongruent stimuli had the shortest reaction times (*M* = 2,979.99 ms, *SD* = 545.19 ms, *Mdn* = 2,913.78 ms; Fig 1B), followed by the slightly incongruent (*M* = 3,131.14 ms, *SD* = 529.98 ms, *Mdn* = 3,031.32 ms; Fig 1B) and congruent stimuli (*M* = 3,236.40 ms, *SD* = 635.06 ms, *Mdn* = 3,097.86 ms; Fig 1B).

**Fig 2:**
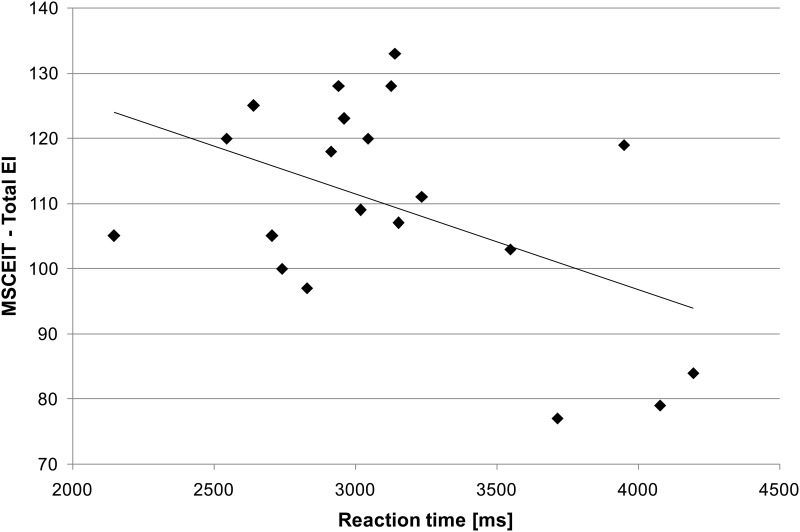
Impact of emotional intelligence on reaction times. Scatterplots illustrate the correlation between total EI and the reaction times for the “ironic” category calculated for slightly incongruent stimuli (*N* = 20, *r* = -.48, *p* = .02, one-tailed).

We found a significant moderately negative correlation between total EI and the mean reaction times for the “ironic” category calculated for the slightly incongruent stimuli (*r* = -.48, *p* = .02, one-tailed; Fig 2). The correlations between total EI and the mean reaction times for the “ironic” category calculated for the congruent (*r* = -.40, *p* = .06, one-tailed) and the strongly incongruent stimuli (*r* = -.23, *p* = .17, one-tailed) did not reach the conventional levels of statistical significance with this sample size but were moderately negative. The correlations between the four branches of the MSCEIT and the mean reaction times for the “ironic” category for all three congruence conditions are summarized in Table 5. Moreover, the correlations between total EI as well as the four branches of the MSCEIT and the mean reaction times for the categories “angry”, “happy”, and “ambivalent” in all three congruence conditions are summarized in Table 5.

**Table 5:** Pearson’s correlation coefficient values between emotional intelligence and the mean reaction times for the four categories across the three congruence conditions.

	MSCEIT				
	Total EI	Perceiving emotions (Branch 1)	Using emotions (Branch 2)	Understanding emotions (Branch 3)	Managing emotions (Branch 4)
*Congruent*					
Angry^2^	-.32	-.42	-.18	-.30	-.07
Happy^2^	-.19	-.18	-.17	-.22	-.01
Ironic^1^	-.40	-.67**	-.19	.01	-.31
Ambivalent^2^	.00	-.16	.07	.12	.04
*Slightly incongruent*					
Angry^2^	-.27	-.36	-.08	-.30	-.14
Happy^2^	-.39	-.43	-.21	-.51*	-.11
Ironic^1^	-.48*	-.41*	-.38*	-.38*	-.32
Ambivalent^2^	-.15	-.22	-.12	.05	-.13
*Strongly incongruent*					
Angry^2^	-.29	-.33	-.17	-.32	-.15
Happy^2^	-.54	-.67*	-.36	-.37	-.39
Ironic^1^	-.23	-.15	-.24	-.09	-.20
Ambivalent^2^	-.15	-.11	-.16	.-10	-.07

MSCEIT = Mayer-Salovey-Caruso Emotional Intelligence Test [20].

^1^ one-tailed.

^2^ two-tailed.

* *p* < .05.

** *p* < .01.

### Nonverbal dominance and emotional intelligence

**Fig 3:**
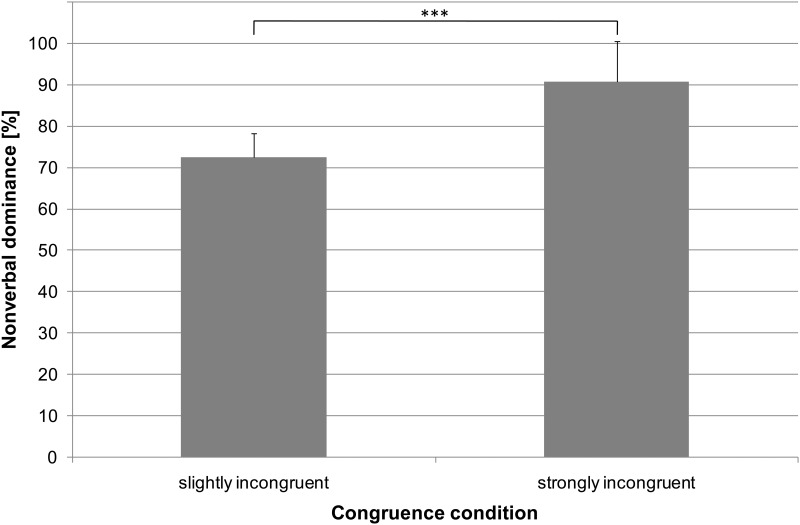
Nonverbal dominance. The bars represent the average nonverbal dominance observed while rating slightly (left bar) and strongly incongruent stimuli (right bar). Error bars represent the standard errors of the means (*N* = 18). Significant differences are marked with asterisks (*** *p* < .001).

For nonverbal dominance calculated for the slightly incongruent stimuli, 72.44% (*SD* = 10.55%, *Mdn* = 69.62%) of the ratings were based on the nonverbal information if one of the emotional categories (“angry”, “happy”) was chosen (Fig 3). When the stimuli were strongly incongruent, this nonverbal dominance was even more pronounced (*M* = 90.82%, *SD* = 11.49%, *Mdn* = 97.50%; *Z* = -3.57, *p* < .001, *r* = -.84, two-tailed; Fig 3).

**Fig 4:**
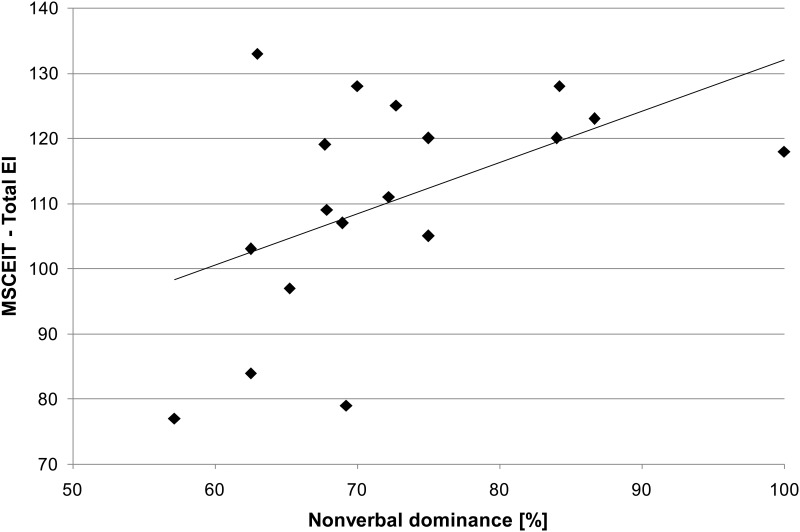
Impact of emotional intelligence on nonverbal dominance. Scatterplots illustrate the correlation between total EI and nonverbal dominance observed while rating slightly incongruent stimuli (*N* = 18, *r*_s_ = .52, *p* = .01, one-tailed).

We found a strong positive correlation between total EI and nonverbal dominance calculated for the slightly incongruent stimuli (*r*_s_ = .52, *p* = .01, one-tailed; Fig 4). No significant correlation was found between total EI and nonverbal dominance calculated for the strongly incongruent stimuli (*r*_s_ = .01, *p* = .48, one-tailed). The correlations between the four branches of the MSCEIT and nonverbal dominance calculated for the slightly and strongly incongruent stimuli are summarized in Table 6.

**Table 6:** Spearman’s rho values between emotional intelligence and nonverbal dominance for the two incongruent conditions.

	MSCEIT				
	Total EI	Perceiving emotions (Branch 1)	Using emotions (Branch 2)	Understanding emotions (Branch 3)	Managing emotions (Branch 4)
*Slightly incongruent*					
NVD^1^	.52*	.39	.46*	.01	.57**
*Strongly incongruent*					
NVD^1^	.01	-.03	.05	-.09	.01

MSCEIT = Mayer-Salovey-Caruso Emotional Intelligence Test [20]; NVD = Nonverbal dominance.

^1^ one-tailed.

* *p* < .05.

** *p* < .01.

## Discussion

The main aim of the study was to evaluate whether (and to what extent) the mismatch between verbal and nonverbal cues would create the impression of irony in the perceiver even in the absence of any further contextual information and to determine how this would be related to perceivers’ EI. We hypothesized that incongruent stimuli would be perceived as “ironic” more often than congruent stimuli. This hypothesis was confirmed: Slightly incongruent stimuli were rated as “ironic” about two times more often than congruent stimuli. For strongly incongruent stimuli, the ratio was 5:1. It is important to note that the actors were not instructed to present irony when acting incongruently. Thus, our findings indicate that the mismatch between verbal and nonverbal information as a reflection of the speaker’s current emotional state created the impression of irony.

With regard to emotional competencies influencing the impression of irony, we hypothesized that EI would be positively associated with the tendency to classify incongruent stimuli as “ironic”. Contrary to this hypothesis, we did not find a significant association between EI and the tendency to classify incongruent stimuli as “ironic”. A possible explanation for this might be that a ceiling effect reduced the effect, and thus, a larger sample size would be needed to detect the impact of EI on choice ratings. Moreover, we hypothesized that EI would be negatively associated with the reaction time needed to classify incongruent stimuli as “ironic”. This hypothesis was partially confirmed: When the stimuli were slightly incongruent, a significant negative association between EI and the mean reaction times was found, indicating that participants higher in EI were faster at rating such stimuli as “ironic” than participants lower in EI. This finding is in line with our previous results, which showed that higher EI was related to smaller reaction time differences between emotionally incongruent and congruent stimuli [3]. Hence, EI seems beneficial for dissolving the irritating conflict between verbal and nonverbal cues more quickly, resulting in a faster grasping of the implicit message. Moreover, in reference to a recent study by Filik and colleagues [25], which suggested that ironic messages may have an influence on the listener’s emotional response, EI might also be helpful for better perceiving, using, understanding, and managing the emotional responses elicited by ironic messages, enabling quicker responses. When the stimuli were strongly incongruent, the task may have been easy enough to mask the effects of EI.

Building on the results of our previous study [3], we hypothesized that when stimuli were incongruent, participants would base their judgment predominantly on the nonverbal part of the message if they chose an emotional category. This hypothesis was confirmed: For both slightly and strongly incongruent stimuli, nonverbal dominance was observed, indicating that participants relied more on the nonverbal part of the message when confronted with conflicting verbal and nonverbal information. Nonverbal dominance was much more pronounced when the stimuli were strongly incongruent. Overall, these results underscore the importance of nonverbal signals in emotional communication processes and their superiority over verbal cues. This frequently observed nonverbal dominance in human communication might be the result of human language development: From a phylogenetic viewpoint, some theories of language development presume that human communication began with the use of nonverbal expressions, and thus, humans “are inherently programmed to attend first and foremost to nonverbal signals” ([26], p. 6). From an ontogenetic viewpoint, infants begin to communicate with nonverbal cues (e.g., facial expressions, pointing gestures, nonverbal vocalizations such as crying) and thus, “[t]he importance of nonverbal modes of expression at this critical and vulnerable stage of life may contribute to our continued dependence on them even though we acquire more sophisticated means of expression” ([26], p. 6). Moreover, we hypothesized that when stimuli were incongruent, EI would be positively associated with the aforementioned nonverbal dominance. This hypothesis was partially confirmed: When stimuli were slightly incongruent, a significant positive association between nonverbal dominance and EI was found, indicating that participants higher in EI relied more on nonverbal cues than participants lower in EI. Individuals with higher EI might have greater knowledge about the relevance of nonverbal signals with respect to implicitly addressing, for instance, the speaker’s true feelings, intentions, and attitudes, and might thereby give more weight to them. When stimuli were strongly incongruent, nonverbal dominance approximated its maximum of 100%. Thus, a ceiling effect may have prevented the expected association between nonverbal dominance and EI with the result that only when the stimuli were slightly incongruent—when the conflicting information was harder to detect—did EI have a measurable positive impact on the ratings.

Regarding the limitations of this study, the following points have to be taken into account: First, the sample size of 20 participants was fairly small, and the group was quite homogenous (e.g., age range, educational level), which restricted the generalizability of the reported results. Second, we provided the category “ironic” but no other forms of non-literal speech (e.g., sarcasm). Thus, no distinction between different forms of non-literal speech could be made. We suggest that future studies offer a larger variety of response options related to such dialogic functions (i.e., “sarcastic”, “dishonest”). Third, offering participants the category “ironic” might have led participants to anticipate that the research focus lay on irony and that the researchers might want them to use this category. Still, the category was only one of four, and the same argument could be made with respect to the impression of ambivalence or the basic emotions. Nevertheless, future studies could use more response options to alleviate such anticipations. Fourth, we could not rule out the possibility that even though the actors were not instructed to produce ironic stimuli, the obvious mismatch between the verbal and nonverbal information or the scenarios used to arouse the actors’ emotions might have accidentally led to nonverbal ironic expressions [13–15]. Therefore, in an additional study, it would be interesting to present the stimulus material to a sample of non-German speakers and to test whether any of the stimuli were categorized as “ironic” due to an ironic facial expression or tone of voice. Fifth, the present study cannot provide an answer to the question of the sequence of neurobiological and cognitive processing during irony perception (e.g., parallel vs. serial processing of verbal and nonverbal cues). In future research, it would be useful to test our paradigm while assessing electrophysiological measures (e.g., EEG) and functional imaging data (e.g., fMRI) to thus offer a better understanding of the processing of irony at a neurobiological level [27].

## Conclusions

The findings of the current study indicate that the mismatch between verbal and nonverbal information conveyed by a speaker creates the impression of irony even in the absence of any further contextual information, with participants high in EI reacting faster. Moreover, the predominant influence of nonverbal cues on ratings of a speaker’s emotional state as well as a positive association between EI and nonverbal dominance was confirmed. Future research should apply the paradigm to patient groups showing deficits in irony processing (e.g., patients diagnosed with autism spectrum disorder or schizophrenia) to examine whether such patients differ from the general population in their tendency to classify the mismatch between verbal and nonverbal information as “ironic”.
